# Supplementary material for: Effect of Praziquantel Treatment on the Nutritional Status of Children Infected with Schistosoma haematobium
Source: Pathogens. 2025 Jan 29;14(2):123. doi: 10.3390/pathogens14020123 (PMC11858091; doi:10.3390/pathogens14020123)
Supplement: Supplementary file 1 [file pathogens-14-00123-s001.zip › pathogens-3374624-supplementary.pdf]

Supplementary Table: Baseline demographic and nutritional characteristics of the viable study participants who were lost to follow-up

		**Treatment group**		**Total**	**P**
		**Praziquantel (infected)**	**Control (uninfected)**		
Sex	Female	29 (14%)	181 (86%)	210	0.21
	Male	47 (19%)	206 (81%)	253	
Age	5 – 10	48 (16%)	256 (84%)	304	0.69
	11 – 15	28 (18%)	131 (82%)	159	
Village	Andada	0 (0%)	26 (100%)	26	<0.01
	Abaro	39 (34%)	77 (66%)	116	
	Buri	2 (7%)	27 (93%)	29	
	Erinbirta	1 (6%)	15 (94%)	16	
	Gabole	2 (17%)	10 (83%)	12	
	Haledebe	2 (11%)	16 (89%)	18	
	Iore	2 (9%)	21 (91%)	23	
	Kalat	0 (0%)	10 (100%)	10	
	Kusara	3 (14%)	18 (86%)	21	
	Mataka	0 (0%)	67 (100%)	67	
	Mender17	6 (7%)	76 (93%)	82	
	Office	0 (0%)	1 (100%)	1	
	Rebada	2 (17%)	10 (83%)	12	
	Tegni	17 (57%)	13 (43%)	30	
Region	Afar	14 (6%)	221 (94%)	235	<0.01
	Gambella	62 (27%)	166 (73%)	228	
Underweight	Yes	40 (34%)	70 (66%)	119	<0.01
	No	36 (10%)	308 (90%)	344	
Wasted*	Yes	10 (32%)	21 (68%)	31	0.02
	No	38 (14%)	235 (86%)	273	
Stunted	Yes	6 (11%)	48 (89%)	54	0.33
	No	70 (17%)	339 (83%)	409	
Mean MUAC		19.32	17.67		0.37
Mean BAZ		–1.94	–1.02		0.01
Mean WAZ*		–1.14	–0.55		<0.01
Mean HAZ		–0.12	–0.46		0.04
Total		76 (16%)	387 (84%)	463	

*Calculations for WAZ and wasting status only included children 10 years of age or younger
